# Yoga as a form of leisure-time physical activity and pregnancy health outcomes

**DOI:** 10.1186/s12884-026-08659-4

**Published:** 2026-02-06

**Authors:** Alexis Thrower, Sarah Modlin, Kara M. Whitaker, Bethany Barone Gibbs

**Affiliations:** 1 Division of Exercise Physiology, West Virginia University, 64 Medical Center Drive, P.O. Box 9216, Morgantown, WV 26506 USA; 2 Department of Epidemiology and Biostatistics, West Virginia University, 64 Medical Center Drive, P.O. Box 9216, Morgantown, WV 26506 USA; 3 Department of Health and Human Physiology, Department of Epidemiology, University of Iowa, 225 S. Grand Avenue, Field House E116, Iowa City, IA 52242 USA

**Keywords:** Pregnancy, Yoga, Physical activity, Cardiovascular, Adverse pregnancy outcome, Gestational weight gain

## Abstract

**Background:**

Leisure-time physical activity (LTPA) promotes pregnancy health, but few studies have investigated how yoga — a mind-body activity — is associated with pregnancy outcomes.

**Objective:**

To determine if engaging in yoga was associated with lower risks of adverse pregnancy outcomes (APO).

**Methods:**

The nuMoM2b prospective cohort study enrolled nulliparous women in their first trimester of pregnancy from eight U.S. clinical centers between October 2010-September 2013 and followed through delivery. LTPA was determined each trimester from an interview querying usual participation in up to three activities (including yoga). Yoga durations were averaged across trimesters. Participants were classified as: no yoga (0 min/week), low yoga (1-<30 min/week), some yoga (30-<75 min/week), or high yoga (≥ 75 min/week). APOs (hypertensive disorders of pregnancy, gestational diabetes, preterm birth (PTB), small-for-gestational age infants) and gestational weight gain (GWG) were prospectively assessed from medical records. Generalized logistic models calculated the risk ratio of outcomes by yoga group, adjusted for sociodemographic, lifestyle, and clinical covariates.

**Results:**

Participants (*n* = 7,502) in the low/some/high yoga groups tended to be older, White, with higher socioeconomic status, and healthier lifestyle behaviors (all *p* < 0.001). Compared to no yoga in fully adjusted models, some and high yoga were associated with fewer APOs (RR = 0.86, 95%CI = 0.76–0.98; RR = 0.78, 95%CI = 0.63–0.96), and specifically PTB for high yoga (RR = 0.42, 95%CI = 0.20–0.87). Low yoga was associated with less inadequate GWG (RR = 0.51, 95%CI = 0.35–0.76) and excessive GWG (RR = 0.69, 95%CI = 0.55–0.86). Some yoga was associated with less excessive GWG (RR = 0.71, 95%CI = 0.57–0.88). Yoga was not associated with other outcomes.

**Conclusions:**

Prenatal yoga may help to protect against some APOs and promote healthy GWG.

**Supplementary Information:**

The online version contains supplementary material available at 10.1186/s12884-026-08659-4.

## Background

Adverse pregnancy outcomes (APOs), such as hypertensive disorders of pregnancy (HDP), preterm birth (PTB), gestational diabetes (GDM), and small-for-gestational age infants (SGA), occur in ~ 20% of pregnancies in the United States [1]. Inappropriate gestational weight gain (GWG) is also highly prevalent, with 20.9% of women experiencing inadequate GWG and 47.2% of women experiencing excessive GWG [2]. One of the few evidence-based methods to improve these pregnancy health outcomes is to engage in leisure-time physical activity (LTPA). LTPA is safe with numerous health benefits when pregnant individuals meet the recommended guidelines of 150–300 min of moderate-intensity physical activity each week, with further health benefits when muscle-strengthening activities are performed at least two days a week [3–5]. The benefits of LTPA during pregnancy are demonstrated by an umbrella review that found strong evidence suggesting that engagement in LTPA during pregnancy reduced the risk of HDP, GDM, and excessive GWG with no extra risk of SGA [6]. This same review did not suggest a relationship between LTPA and other pregnancy outcomes (e.g., PTB and inadequate GWG).

Yoga is an increasingly popular LTPA, including during pregnancy, as it is low impact, intensity can be adjusted, and it requires minimal equipment/space [3, 7, 8]. This is supported by the Nulliparous Pregnancy Outcomes Study: Monitoring Mothers-to-Be (nuMoM2b) cohort study, where yoga was the third most commonly reported LTPA [9]. Prenatal yoga may be popular because it can help to overcome some of the commonly reported barriers to achieving LTPA guidelines during pregnancy including fatigue, time constraints, pregnancy symptoms/discomforts (e.g., nausea), safety concerns related to higher physical activity intensity, and a lack of access to facilities/affordability [3, 10]. 

Moreover, yoga may have benefits that could improve pregnancy health beyond those traditionally achieved through LTPA, as yoga is a mind-body practice combining three components: yoga poses (*asanas*), breathing practices (*pranayama*), and mindfulness/meditation (*sati and dhyana*) [11]. Interventions that included only breathing and meditation have been found to benefit cardiovascular responses in non-pregnant populations, for example lowering blood pressure [12, 13]. The additional mind-body components of yoga may be uniquely beneficial as a form of LTPA during pregnancy that can also contribute toward meeting weekly LTPA guidelines independently or in combination with other activities. Despite the potential benefits from yoga, very few large observational studies or randomized controlled trials have investigated yoga during pregnancy. Among those available, limitations exist such as lack of adjustment for important confounders, not considering dose-response associations, not considering GWG as an outcome in observational studies, and small and/or less generalizable samples in clinical trials [14–17]. More rigorous research clarifying associations between yoga and pregnancy outcomes is needed to better understand yoga’s impact.

The purpose of this secondary analysis was to determine if pregnant individuals who self-reported engaging in yoga during pregnancy had better pregnancy outcomes compared to those who did not participate in yoga (both with/without accounting for other LTPA). Pregnancy outcomes included any APO, inclusive of HDP, PTB, GDM, and SGA, and GWG in clinically defined categories (inadequate, adequate, or excessive). We hypothesized that yoga participation during pregnancy would be associated with better pregnancy outcomes and that benefits would be observed even after accounting for engagement in other LTPA.

## Methods

This is a secondary analysis of the nuMoM2b prospective cohort study. Detailed methods for nuMoM2b are reported previously [18] and briefly below.

### Participants

**Fig. 1:**
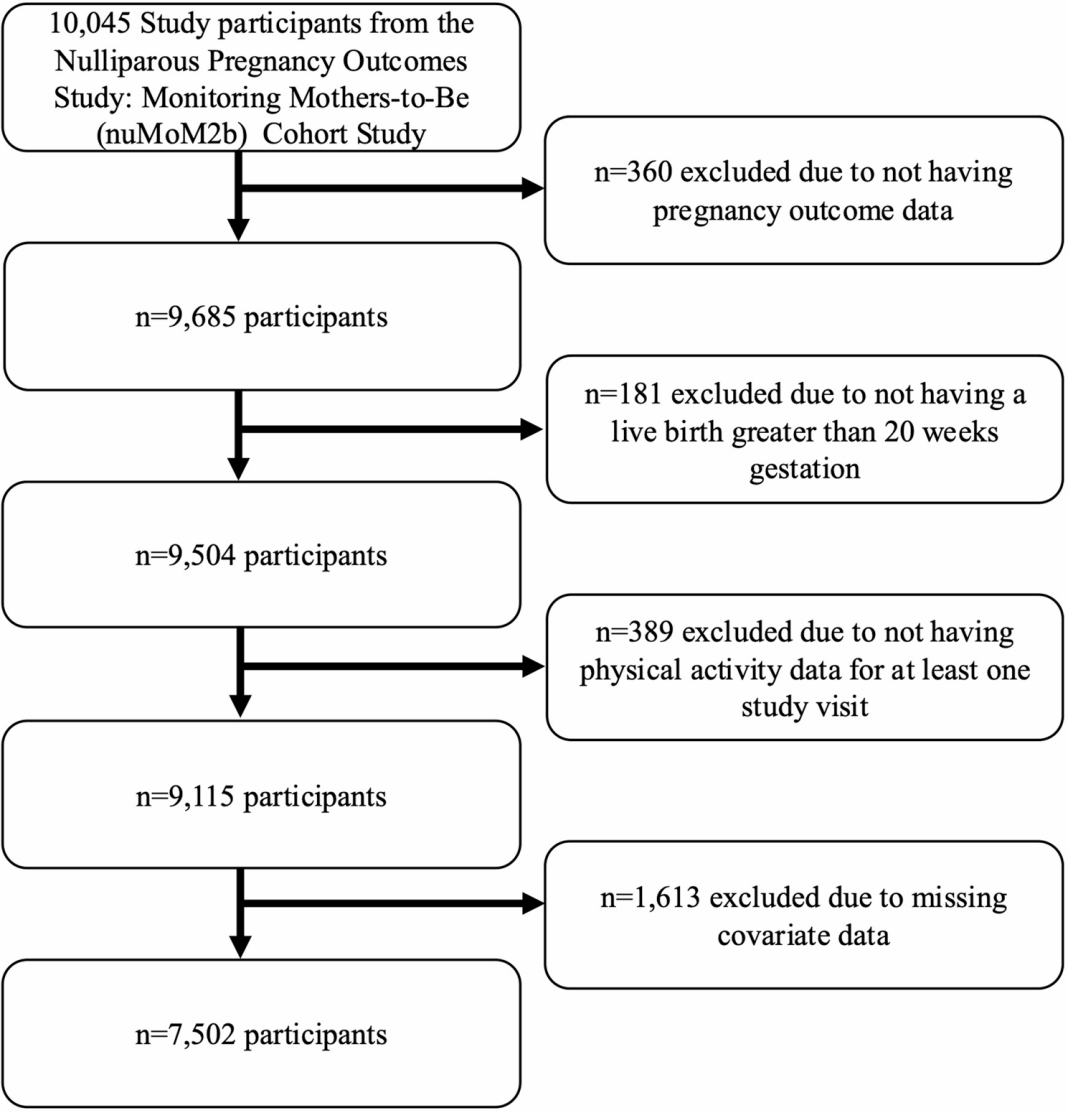
Flowchart of Participant Exclusion Criteria for Analytic Sample. This flowchart displays the sample sizes and reasons for excluding participants from this secondary analysis

10,045 nulliparous pregnant individuals were recruited (October 2010-September 2013) from eight sites across the United States (Case Western University, Columbia University, Indiana University, Northwestern University, University of California at Irvine, University of Pennsylvania, University of Pittsburgh, and University of Utah). To be eligible, participants had to have a viable singleton pregnancy, no prior pregnancy > 20 weeks, and gestational age between 6 and 13 weeks. Participants attended three in-person study visits across pregnancy (between 6 and < 14 weeks gestation, between 16 and < 22 weeks gestation, and between 22 and < 30 weeks gestation) for interviews and clinical assessments. For the current analysis, participants were excluded if outcome data were missing, if pregnancy ended at < 20 weeks gestation, if LTPA data were not available for at least one study visit, or if covariate data were missing (see Fig. 1). Of the 10,045 participants, 7,502 were included in analyses, with missing dietary data as the most common reason for exclusion (7%). Participants included/excluded from analyses are compared in Supplemental Table 1; those included in our analytic sample were generally older, with higher education and income, reported non-Hispanic White race, had private health insurance, and generally had healthier lifestyle behaviors, lower pre-pregnancy body mass index (BMI), and greater gestational age at delivery (all *p* < 0.001). Each site’s Institutional Review Board approved research protocols and all participants provided written informed consent. If participants were considered minors in the state they enrolled, informed consent was obtained from their legal guardian, and assent was obtained from the minor.

### Participant characteristics

Most participant characteristics were recorded during an interview at the first study visit. Participants reported maternal age, education, family income, insurance status, and race. Alcohol and tobacco use within one month prior to the visit was reported during the first, second, and third trimester visits and at delivery. If participants reported yes to alcohol or tobacco from any of the four visits, they were considered to have utilized these substances for our analyses. Early pregnancy BMI was determined by height and weight converted to kg/m^2^. Diet was measured by the modified Block 2005 Food Frequency Questionnaire [19] and scored using the Healthy Eating Index 2010 [20]. This method is reliable (Cronbach’s α value = 0.68) [21] and higher scores for the Healthy Eating Index values indicated healthier diets. As we predetermined diet would be an important confounder between yoga and pregnancy outcomes, we excluded participants missing this important covariate.

### Yoga and other leisure-time physical activity (LTPA)

Participation in yoga and other forms of LTPA was determined from self-reported responses to questions adapted from the reliable Behavioral Risk Factor Surveillance Survey (BRFSS) at the three visits across pregnancy [22, 23]. Participants were asked to report participation in any physical activities/exercises in the past four weeks. Then, through open-ended responses, they reported up to three types of activity (including yoga, if applicable), the number of times per week they engaged in each activity, and the average minutes spent performing the activity during each session.

If participants reported any type of yoga in their response to the LTPA questions during the first, second, or third trimester study visit, then they were considered to have done yoga during pregnancy. Weekly duration of yoga was calculated by multiplying the reported minutes of yoga by days per week for each visit. Participants who did not report engaging in yoga as a response to the LTPA questions were assigned a yoga duration value of 0 for that visit. A total pregnancy yoga duration variable was calculated by averaging durations of yoga reported across all study visits (up to three). If data was missing for any study visit, an average of the available yoga durations was used to estimate the overall average yoga duration. For analyses evaluating associations between yoga participant and outcomes, four yoga groups were determined based on the overall average: no yoga (0 min/week), low yoga (1 to < 30 min/week), some yoga (30 to < 75 min/week), or a high yoga group (≥ 75 min/week).

LTPA for activities (except yoga) with intensities corresponding to at least moderate intensity (3-5.9 metabolic equivalents (METs)) or vigorous intensity (≥ 6.0 METs) were converted to durations (minutes/week) for all study visits, consistent with current Physical Activity Guidelines for Americans [3]. Although some styles of yoga can be considered a form of moderate-intensity LTPA [24], we did not include yoga durations into our LTPA calculation as we hoped to investigate the independent effects of yoga and other LTPA participation. As above, minutes spent engaging in each activity were multiplied by the number of days per week for that activity to calculate weekly durations. Participants that indicated no physical activities were assigned a value of 0 min/week. Weekly LTPA was converted to total moderate-equivalent minutes by summing weekly durations of moderate intensity activities and 2 x weekly durations of vigorous intensity activities within each study visit [3]. An overall pregnancy LTPA duration was calculated by averaging moderate-equivalent minutes across all available study visits (up to three). Participants were classified into one of four physical activity guideline groups based on their average activity across pregnancy, as follows: none or no physical activity per week (0 min/week), insufficient physical activity (between 1 and 149 min/week), sufficient physical activity (150–299 min/week), or high physical activity (300 min/week or greater).

### Pregnancy outcomes

Maternal and infant outcomes were abstracted prospectively from medical records by a certified abstractor 30 days after delivery. The following APOs were included in this secondary analysis as binary variables: HDP, PTB, GDM, and SGA. HDP included gestational hypertension, preeclampsia with/without severe features, super-imposed preeclampsia, and eclampsia based on standard criteria [25]. PTB was defined as delivery prior to 37 weeks gestion, and GDM was defined by Whites classification of abstracted glucose tolerance testing data [25]. SGA was based on the Alexander fetal growth curve standards where infants < 10th percentile of infant birthweight for their gestational age were classified as having SGA [26]. Finally, participants were classified as experiencing an APO if they had one or more of these outcomes.

GWG across pregnancy was calculated as the difference between maternal weight at delivery and self-reported pre-pregnancy weight. Participants were classified into GWG categories based on their pre-pregnancy BMI and the Institute of Medicine guidelines [27]. Classifications included inadequate GWG, adequate GWG, or excessive GWG.

### Statistical analyses

Stata BE version 17.0 (StataCorp LLC, College Station, TX, USA) was used for statistical analyses with the two-sided significance level set to *p* < 0.05. One-way ANOVA or chi-square tests determined differences in participant characteristics across groups. General logistic regression models calculated the relative risk (RR) of APOs by categorical yoga group with no yoga as the reference group. Participants were excluded from HDP regression models if they had hypertension prior to pregnancy (*n* = 180) and from GDM regression models if they had diabetes mellitus prior to pregnancy (*n* = 109). Multinomial logistic regression models calculated the RR of GWG categories by categorical yoga group, with the no yoga group and adequate GWG acting as the independent and dependent variable reference groups, respectively. Each model was refit with yoga group as an ordinal variable (rather than categorical) to evaluate p-trend with increasing yoga participation.

Three models were analyzed for each APO: (1) unadjusted, (2) adjusted for possible confounders including age, early pregnancy BMI, income, insurance, race, diet, any prenatal alcohol use, and any prenatal tobacco use; and (3) adjusted for model 2 covariates and LTPA, to evaluate benefits of yoga beyond LTPA. These same three models were used for GWG outcomes, except early pregnancy BMI was excluded from these analyses due to BMI being utilized to calculate GWG. Also, gestational age at delivery was an additional confounder considered for the GWG models. Education was considered for covariate adjustment but was not included due to collinearity. Age was converted to a categorical variable for analyses due to collinearity when included as a continuous variable. The age group categories were chosen based on an increased risk of pregnancy complications being associated with younger and advanced maternal age [28, 29]. Due to convergence issues for the model predicting composite APOs, age, income, and race were excluded as covariates; sensitivity analyses where these covariates were added individually to the models did not affect the association between yoga and composite APO, suggesting adjustment for age, income, and race would have not influenced our reported associations.

Two sensitivity analyses were conducted to evaluate the robustness of our findings. The first repeated fully adjusted models without diet as a covariate, allowing for inclusion of the substantial proportion of participants who were excluded due to missing diet. Additionally, consistency of participating in yoga across pregnancy may be more important than weekly durations of yoga. Thus, we repeated fully adjusted models based on 3 yoga exposure groups defined as: None (no yoga in any trimester), Inconsistent (yoga is reported in some but not all available trimesters), and Consistent (yoga is reported in all available trimesters).

## Results

Table 1 presents participant characteristics overall and by yoga group. Greater yoga participation was generally associated with different participant demographics including older age, higher education and income, being non-Hispanic White, and having private health insurance (all *p* < 0.001). Behaviors and clinical characteristics also differed with greater yoga participation typically associated with a healthier diet, being more active, less smoking, more alcohol consumption, lower early pregnancy BMI, and greater gestational age at delivery (all *p* < 0.001).

**Table 1:** Participant characteristics by yoga group

	Overall (*n* = 7,502)	No Yoga (*n* = 6,237)	Low Yoga (*n* = 464)	Some Yoga (*n* = 558)	High Yoga (*n* = 243)	*p*-value
Demographics						
Age Categories						**< 0.001**
13–21 years	1,370 (18.3)	1,322 (21.2)	24 (5.2)	21 (3.8)	3 (1.2)	
22–35 years	5,619 (74.9)	4,531 (72.6)	405 (87.3)	475 (85.3)	208 (85.6)	
> 35 years	513 (6.8)	384 (6.2)	35 (7.5)	62 (10.9)	32 (13.2)	
Education						**< 0.001**
Less than high school graduate	510 (6.8)	500 (8.0)	6 (1.3)	3 (0.5)	1 (0.4)	
High school graduate or GED	817 (10.9)	789 (12.6)	14 (3.0)	12 (2.2)	2 (0.8)	
Some higher education	2,099 (28.0)	1,906 (30.6)	89 (19.2)	77 (13.8)	27 (11.1)	
Bachelor’s degree or higher	4,076 (54.3)	3,042 (48.8)	355 (76.5)	466 (83.5)	213 (87.7)	
Race						**< 0.001**
Non-Hispanic White	4,770 (63.6)	3,799 (60.9)	349 (75.2)	418 (74.9)	204 (83.9)	
Non-Hispanic Black	837 (11.2)	794 (12.7)	16 (3.5)	23 (4.1)	4 (1.7)	
Hispanic	1,215 (16.2)	1,093 (17.5)	51 (11.0)	54 (9.7)	17 (7.0)	
Other	680 (9.0)	551 (8.8)	48 (10.3)	63 (11.3)	18 (7.4)	
Household income						**< 0.001**
Less than 25,000	1,019 (13.6)	949 (15.2)	32 (6.9)	25 (4.5)	13 (5.4)	
$25,000- $49,999	934 (12.4)	839 (13.5)	36 (7.8)	39 (7.0)	20 (8.2)	
$50,000-$99,999	1,926 (25.7)	1,555 (24.9)	131 (28.2)	167 (29.9)	73 (30.0)	
$100,000 or above	2,375 (31.7)	1,722 (27.6)	232 (50.0)	291 (52.2)	130 (53.5)	
Not reported	1,248 (16.6)	1,172 (18.8)	33 (7.1)	36 (6.4)	7 (2.9)	
Insurance type						**< 0.001**
Public	1,781 (23.7)	1,690 (27.1)	47 (10.1)	34 (6.1)	10 (4.1)	
Private	5,583 (74.4)	4,424 (70.9)	412 (88.8)	518 (92.8)	229 (94.2)	
Not reported	138 (1.8)	123 (2.0)	5 (1.1)	6 (1.1)	4 (1.7)	
Behaviors						
LTPA guideline category (yoga excluded as a form of LTPA for this variable)						
No LTPA	902 (12.0)	861 (13.8)	19 (4.1)	17 (3.1)	5 (2.1)	
Insufficient	3,687 (49.2)	3,130 (50.2)	232 (50.0)	230 (41.2)	95 (39.1)	
Sufficient	1,871 (24.9)	1,421 (22.8)	147 (31.7)	213 (38.2)	90 (37.0)	
Exceeding	1,042 (13.9)	825 (13.2)	66 (14.2)	98 (17.5)	53 (21.8)	
Healthy Eating Index						**< 0.001**
Quantile 1	1,876 (25.0)	1,769 (28.4)	52 (11.2)	41 (7.4)	14 (5.8)	
Quantile 2	1,875 (25.0)	1,640 (26.3)	103 (22.2)	96 (17.2)	36 (14.8)	
Quantile 3	1,876 (25.0)	1,498 (24.0)	135 (29.1)	172 (30.8)	71 (29.2)	
Quantile 4	1,875 (25.0)	1,330 (21.3)	174 (37.5)	249 (44.6)	122 (50.2)	
Any prenatal alcohol use						**< 0.001**
No	6,486 (86.5)	5,521 (88.5)	366 (78.9)	419 (75.1)	180 (74.1)	
Yes	1,016 (13.5)	716 (11.5)	98 (21.1)	139 (24.9)	63 (25.9)	
Any prenatal tobacco use						**< 0.001**
No	7,025 (93.6)	5,779 (92.7)	455 (98.1)	550 (98.6)	241 (99.2)	
Yes	477 (6.4)	458 (7.3)	9 (1.9)	8 (1.4)	2 (0.8)	
Clinical Characteristics						
Early pregnancy BMI						**< 0.001**
BMI < 30 kg/m^2^	4,055 (54.1)	3,209 (51.4)	307 (66.2)	360 (64.5)	179 (73.7)	
BMI ≥ 30 kg/m^2^	3,447 (45.9)	3,028 (48.6)	157 (33.8)	198 (35.5)	64 (26.3)	
Gestational age at delivery^a^						**< 0.001**
Gestational age, weeks	39.3 (2.0)	39.2 (2.0)	39.5 (1.6)	39.6 (1.6)	39.6 (1.7)	

Data presented as mean (standard deviation) for continuous variables or n (percent) for categorical variables; Bold p-value indicates statistical significance

BMI Body mass index, LTPA Leisure-time physical activity

^a^indicates sample sizes differed for this variable with missing data by group as follows: *n* = 10 for the no yoga group, *n* = 1 for the some yoga group, and *n* = 1 for the high yoga group

### Pregnancy outcome results

**Fig. 2:**
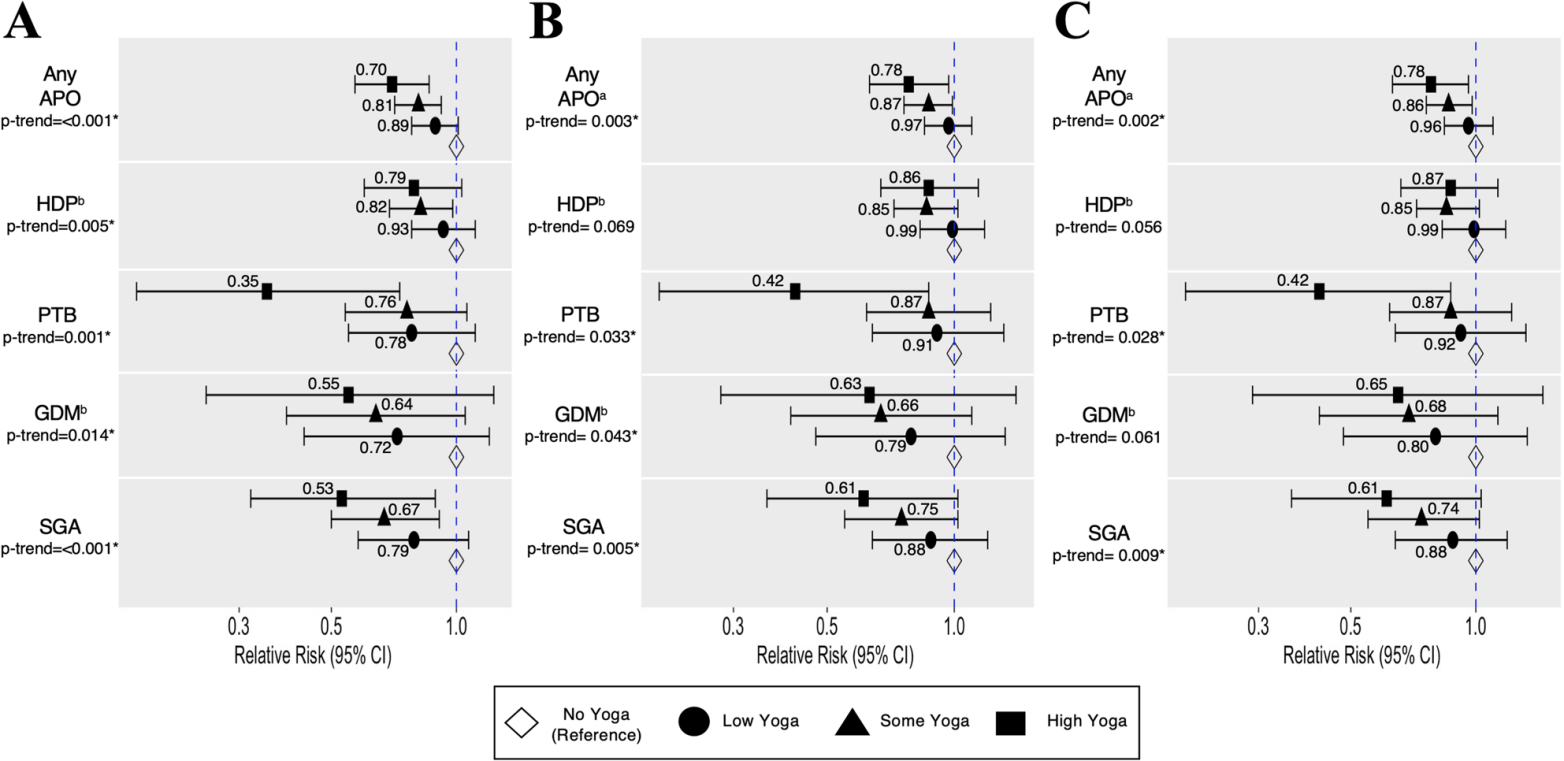
Relative Risk of Experiencing Adverse Pregnancy Outcomes by Yoga Group. Forest plots demonstrate the relative risk and 95% confidence intervals for each APO by general linear model. Panel **A** displays unadjusted results (Model 1). Panel **B** displays results adjusted for age, early pregnancy BMI, income, insurance, race, diet, prenatal alcohol use, and prenatal tobacco use (Model 2). Panel **C** displays results adjusted for Model 2 + LTPA (Model 3); a by an outcome indicates age and income were not included as adjustment variables due to convergence issues within the general linear model; b by an outcome indicates that participants were not included in analyses due to having a preexisting condition prior to pregnancy;*indicates a statistically significant p-trend; Abbreviations: APO=adverse pregnancy outcome, HDP=hypertensive disorders of pregnancy, PTB=preterm birth, GDM=gestational diabetes mellitus, and SGA=small-for-gestational age infant

RR from adjusted models associated low, some, or high yoga vs. no yoga with any APO and each APO individually (Fig. 2; sample sizes and risk estimates are reported in Supplemental Table 2). In unadjusted models (Model 1), both some and high yoga were associated with a decreased risk of SGA, and any APO; only some yoga was associated with lower risk of HDP which may reflect smaller samples sizes in the high yoga group. A significant trend of reduced risk across increasing yoga groups was observed for all APO outcomes, considered together or individually (all *p* < 0.015).

After adjustment for confounders (Model 2), associations were similar though attenuated. Only associations between high yoga and any APO or PTB and between some yoga and any APO remained significant; significant trends of reduced risk with increasing amount of yoga persisted for any APO, PTB, GDM, and SGA (all *p* < 0.05). With additional adjustment for LTPA (Model 3) a significant association was still observed between high yoga and any APO (RR = 0.78, 95%CI = 0.63–0.96) and PTB (RR = 0.42, 95%CI = 0.20–0.87) and between some yoga and any APO (RR = 0.86, 95%CI = 0.76–0.98), though p-trend between yoga and GDM became non-significant.

Table 2 presents RR of inadequate or excessive GWG across groups. Low yoga was associated with a significantly reduced risk of both inadequate and excessive GWG compared to the no yoga and adequate GWG reference group, even in fully adjusted models. Some yoga was associated with a significantly reduced risk of inadequate GWG only in unadjusted models and excessive GWG in all three models. High yoga was not significantly associated with risk of inadequate or excessive GWG outcomes compared to the no yoga and adequate GWG reference group. P-trend only indicated a significantly reduced risk of inadequate GWG with greater yoga engagement in unadjusted models (*p* < 0.001), becoming non-significant with any adjustment (*p* > 0.05); p-trend indicating a significant reduced risk of excessive GWG with greater yoga engagement in all three models (*p* < 0.01).

**Table 2:** Relative risk of Inadequate, Adequate, or excessive gestational weight gain by yoga group

Outcome	Yoga Group	Total *N*	Percent of Events	Model 1 RR (95% CI)	*p*-value	Model 2 RR (95% CI)	*p*-value	Model 3 RR (95% CI)	*p*-value
Inadequate GWG	None	778	13.1%	1.0 (Reference)	-	1.0 (Reference)	-	1.0 (Reference)	-
	Low	35	7.8%	0.42 (0.28–0.61)	**< 0.001**	0.52 (0.35–0.77)	**0.001**	0.51 (0.35–0.76)	**0.001**
	Some	59	10.9%	0.60 (0.44–0.83)	**0.002**	0.78 (0.57–1.09)	0.144	0.77 (0.56–1.07)	0.123
	High	28	12.3%	0.81 (0.50–1.29)	0.366	1.11 (0.68–1.79)	0.666	1.10 (0.68–1.78)	0.694
				**p-trend**	**< 0.001**	**p-trend**	0.188	**p-trend**	0.159
Adequate GWG	None	1,164	19.5%	1.0 (Reference)	-	1.0 (Reference)	-	1.0 (Reference)	-
	Low	126	27.9%	1.0 (Reference)	-	1.0 (Reference)	-	1.0 (Reference)	-
	Some	146	26.9%	1.0 (Reference)	-	1.0 (Reference)	-	1.0 (Reference)	-
	High	52	22.8%	1.0 (Reference)	-	1.0 (Reference)	-	1.0 (Reference)	-
Excessive GWG	None	4,016	67.4%	1.0 (Reference)	-	1.0 (Reference)	-	1.0 (Reference)	-
	Low	290	64.3%	0.67 (0.54–0.83)	**< 0.001**	0.70 (0.56–0.88)	**0.002**	0.69 (0.55–0.86)	**0.001**
	Some	338	62.3%	0.67 (0.54–0.82)	**< 0.001**	0.72 (0.58–0.89)	**0.003**	0.71 (0.57–0.88)	**0.002**
	High	148	64.9%	0.82 (0.60–1.14)	0.242	0.90 (0.65–1.25)	0.525	0.89 (0.64–1.24)	0.508
				**p-trend**	**< 0.001**	**p-trend**	**0.003**	**p-trend**	**0.002**

Model 1 was unadjusted

Model 2 was adjusted for age, income, insurance, race, diet, prenatal alcohol use, prenatal tobacco use, and gestational age at delivery

Model 3 was adjusted for Model 2 + LTPA

RR Relative risk, 95 % CI 95 % confidence interval, GWG Gestational weight gain

Bold p-value indicates statistical significance

Supplemental Table 3 reports results from the sensitivity analyses including participants with missing diet data. Risk estimates and statistical significance remained similar across all outcomes, except HDP, where a significant reduction in risk was now observed for the some yoga group compared to no yoga (RR = 0.81, 95% CI = 0.69–0.96) and the p-for-trend became significant. Conversely, a statistically significant reduction in PTB risk was no longer present for the high yoga group (though the p-for-trend remained significant). Sensitivity analyses included yoga groups that represented consistency of yoga across pregnancy, rather than the weekly duration. Overall, findings with these yoga consistency groups were similar to our original weekly duration yoga groups in that any yoga participation was protective against APOs and inappropriate GWG (see Supplemental Table 4).

## Discussion

This secondary analysis examined associations between engaging in different durations of yoga throughout pregnancy and pregnancy health outcomes. Compared to those who did not self-report engaging in yoga, yoga participation was suggestive of improvements in experiencing any APO, especially PTB. Low participation in yoga was associated with both less inadequate GWG and less excessive GWG, while some yoga was associated with less excessive GWG. Higher weekly durations of yoga (at least 75 min/week) appear to be necessary to significantly reduce the risk of experiencing an APO like PTB. Although not significant in fully adjusted models, favorable reductions in risk were observed for HDP, GDM, and SGA, especially in the high yoga group, and non-significantly lower risks were observed for inadequate GWG and excessive GWG with higher weekly engagement in yoga. Overall, those who self-reported engaging in yoga during pregnancy appeared to have better pregnancy health outcomes.

A unique finding of this secondary analysis was that yoga engagement was associated with a reduced risk of PTB after adjusting for other forms of LTPA. Despite the available evidence suggesting no association between non-yoga physical activity and these APOs [6], our findings suggest that yoga as a mind-body activity may provide additional benefits toward these outcomes beyond a training and conditioning effect. Additionally, our conclusions complement those of a brief research letter published in October of 2024 by an independent research group using the same cohort that similarly found any yoga during the first trimester of pregnancy was associated with lower odds of adverse pregnancy outcomes (especially PTB); we meaningfully expand upon this letter by reporting progressively adjusted models, considering different levels of yoga participation, adjusting for diet, and evaluating associations of yoga with GWG [27]. Yoga may provide benefits to pregnancy health through stress reduction; specifically, previous studies have established an association between stress during pregnancy with PTB and SGA [31, 32]. Although our fully adjusted models only found significantly lower risk comparing high to no yoga for PTB, p-trend values suggest an association between greater yoga and lower risk for PTB and SGA. In non-pregnant adults, yoga reduces stress by improving several physiological markers of stress (e.g., cortisol and blood pressure) [33]. A recent meta-analysis in pregnant individuals determined that stress-reducing interventions (six of 10 included yoga) implemented during pregnancy significantly reduced the risk of PTB (RR = 0.50, 95% CI = 0.35–0.71) [34]. Further, a systematic review reported that yoga interventions performed during pregnancy improved subjective stress measurements [35]. Taken together, our findings indicate that yoga engagement during pregnancy may lead to lower the risk of PTB and SGA, and yoga-related stress reduction is a plausible pathway to this benefit.

In contrast, though several pregnancy studies have observed improvements in HDP, GDM, and related cardiometabolic risk factors (e.g., blood pressure and blood glucose levels) with yoga participation [36, 37], we did not observe significantly reduced risk in these outcomes in fully adjusted models (though risk estimates were in the protective direction). Our findings differ from those of traditional supervised exercise trials that have observed lower risk of HDP and GDM but not other APOs that yoga seemed to benefit in our analyses (e.g., PTB) [6]. A strength of our analyses was considering weekly duration of yoga, but we were unable to determine yoga intensity. This is likely an important factor for decreasing HDP and GDM, since the exercise trials demonstrating reduced risks of these outcomes are typically of moderate intensity or greater. Additionally, participants in our some yoga group and most of participants in the high yoga group (~ 80%) were not meeting LTPA guidelines of 150–300 min/week through yoga alone. Thus, our findings suggest that engagement in varying yoga durations in this cohort appeared to have different and complimentary benefits than those expected from a traditional exercise intervention. Future research could test whether yoga interventions of higher intensity that also achieve LTPA guidelines (such as vinyasa yoga) might result in benefits to PTB (as observed here) along with HDP and GDM (that are consistently reduced in exercise training studies).

We observed a reduced risk of inadequate and excessive GWG for low vs. no yoga and for excessive GWG with some vs. no yoga in our fully adjusted models. This is an important contribution to the literature since we are unaware of other studies testing similar hypotheses. Our findings harmonize with studies in non-pregnant adults that observed weight loss from yoga engagement [38], suggesting similar health benefits to other common forms of LTPA. Our risk reduction of ~ 30% in adjusted models comparing some vs. no yoga is similar to a meta-analysis by Ruchat et al. investigating the effect of exercise interventions on GWG. These authors found a pooled risk reduction of 32% (OR = 0.68; 95% CI = 0.59–0.78) for the risk of excessive GWG based on the results of 32 studies [39]. However, we did not observe a statistically significant benefit in fully adjusted models for our high yoga group. Considering that p-trend values indicated benefit for this outcome with any yoga participation, we can speculate that the smaller sample size and potentially residual confounding may have contributed to these non-significant results. Contrary to our additional favorable findings of ~ 49% reduction in the risk of inadequate GWG with low vs. no yoga participation, the same meta-analysis mentioned above observed a 22% (OR = 0.88; 95% CI = 1.03–1.45) increase in the risk of inadequate GWG based on pooled estimates from 12 exercise interventions. This discrepancy between our finding and this meta-analysis for inadequate GWG may be due to low quality included studies, the higher volume of engagement reported within the meta-analysis (averaged 550 MET-minutes/week), and the meta-analysis focused on exercise rather than yoga during pregnancy [39]. Future trials that report yoga frequency, duration, and intensity as well as other types of physical activity during pregnancy may enhance our understanding yoga’s impact on GWG.

## Strengths, Limitations, and Future directions

Several strengths of this secondary analysis include the large, representative US pregnancy cohort with varying levels of LTPA and yoga, adjustment and sensitivity analyses for important potential confounding variables (e.g., diet), sensitivity analyses considering yoga consistency, and investigation of several pregnancy health outcomes. Additionally, yoga participation was prospectively assessed across pregnancy and high-quality pregnancy outcomes came from medical records abstracted by trained professionals. Limitations include the observational study design which, despite our careful consideration and adjustment for potential confounders (except for occupation, which was not available), remains vulnerable to residual confounding and limits causal inference. Also, yoga and LTPA were self-reported and subject to response bias, though a standard, reliable questions were used. Additionally, we were not able to evaluate types or components of yoga due to limitations of the data collection instrument. Future observational studies querying detailed yoga information could help to clarify associations between types of yoga and pregnancy health outcomes, and experimental studies with specific yoga protocols performed throughout pregnancy will provide the most rigorous evidence. Future research should explore the relationship between yoga and stress in pregnancy as a pathway to improved APOs.

## Conclusion

The results of this secondary analysis suggest that yoga participation during pregnancy may protect against the development of APOs, especially PTB, even after accounting for usual LTPA level. Further, participation in low or some yoga may promote a healthy GWG. As yoga participation was never associated with worse pregnancy outcomes, we can generally conclude that engaging in yoga during any trimester of pregnancy had better pregnancy outcomes compared to those who did not engage in yoga. Especially considering that yoga appeared to have unique benefits to PTB not usually observed with standard exercise training interventions, our promising findings strongly support future experimental trials with rigorous yoga protocols to confirm and better understand the possible pregnancy-related benefits of yoga.

## Supplementary Information

Supplementary Material 1.

Supplementary Material 2.

Supplementary Material 3.

Supplementary Material 4.

## Data Availability

The datasets used and/or analysed during the current study (the Nulliparous Pregnancy Outcomes Study: Monitoring Mothers-to-Be dataset) are available in the National Institutes of Health Eunice Kennedy Shriver National Institute of Child Health and Human Development Data and Specimen Hub (DASH) repository,(https:/dash.nichd.nih.gov).

## Abbreviations

LTPA: Leisure-time physical activity
nuMoM2b: Nulliparous Pregnancy Outcomes Study: Monitoring Mothers-to-Be
APO: Adverse pregnancy outcome
HDP: Hypertensive disorders of pregnancy
PTB: Preterm birth
GDM: Gestational diabetes
SGA: Small-for-gestational age infants
GWG: Gestational weight gain
BMI: Body mass index
RR: Relative risk

## Declarations

### Author’s information

AT holds a MS in Exercise Physiology and is currently a PhD candidate in the Division of Exercise Physiology at West Virginia University (Morgantown, WV). Additionally, AT has earned two separate 200-hour registered yoga teacher certifications. SM holds an MPH in Data Analytics and is a research associate at West Virginia University (Morgantown, WV). KW is an Associate Professor and Director of Graduate Studies at the University of Iowa (Iowa City, IA) with an MPH in Physical Activity and PhD in Exercise Science. BBG is a Professor and Chair of the Epidemiologist and Biostatistics Department at West Virginia University (Morgantown, WV) with an ScM and PhD in Epidemiology.
