# Supplementary material for: Yoga as a form of leisure-time physical activity and pregnancy health outcomes
Source: BMC Pregnancy Childbirth. 2026 Feb 6;26:252. doi: 10.1186/s12884-026-08659-4 (PMC12977424; doi:10.1186/s12884-026-08659-4)
Supplement: Supplementary file 1 — Supplementary Material 1. [file 12884_2026_8659_MOESM1_ESM.docx]

| Supplemental Table 1. Comparison of Participant Characteristics for those Included in the Analytic Sample versus Excluded from Analyses | | | |
| --- | --- | --- | --- |
|  | **Analytic Sample** (n=7,502) | **Excluded from Analyses** (n=2,543) | **p-value** |
| Demographics | | | |
| Age Categories by Year |  |  | **<0.001** |
| 13-21 | 1,370 (18.3) | 763 (30.2) |  |
| 22-35 | 5,619 (74.9) | 1,603 (63.5) |  |
| >35 | 513 (6.8) | 160 (6.3) |  |
| Education |  |  | **<0.001** |
| Less than high school graduate | 510 (6.8) | 306 (12.1) |  |
| High school graduate or GED | 817 (10.9) | 354 (14.1) |  |
| Some higher education | 2,099 (28.0) | 854 (33.9) |  |
| Bachelor’s degree or higher | 4,076 (54.3) | 1,004 (39.9) |  |
| Race |  |  | **<0.001** |
| Non-Hispanic White | 4,770 (63.6) | 1,219 (48.3) |  |
| Non-Hispanic Black | 837 (11.2) | 581 (23.0) |  |
| Hispanic | 1,215 (16.2) | 485 (19.2) |  |
| Other | 680 (9.0) | 241 (9.5) |  |
| Household income |  |  | **<0.001** |
| Less than 25,000 | 1,019 (13.6) | 472 (18.6) |  |
| $25,000- $49,999 | 1934 (12.4) | 370 (14.5) |  |
| $50,000-$99,999 | 1,926 (25.7) | 458 (18.0) |  |
| $100,000 or above | 2,375 (31.7) | 576 (22.7) |  |
| Not reported | 1,248 (16.6) | 667 (26.2) |  |
| Insurance type |  |  | **<0.001** |
| Public | 1,781 (23.8) | 928 (36.7) |  |
| Private | 5,583 (74.4) | 1,532 (60.7) |  |
| Not reported | 138 (1.8) | 66 (2.6) |  |
| Behaviors | | | |
| LTPA guideline category |  |  | **<0.001** |
| None | 902 (12.0) | 428 (20.1) |  |
| Insufficient | 3,687 (49.2) | 940 (44.2) |  |
| Sufficient | 1,871 (24.9) | 422 (19.8) |  |
| High | 1,042 (13.9) | 338 (15.9) |  |
| Healthy Eating Index |  |  | 0.765 |
|  | 63.0 (12.6) | 62.9 (12.7) |  |
| Any prenatal alcohol use |  |  | **<0.001** |
| No | 6,486 (86.5) | 2,233 (90.8) |  |
| Yes | 1,016 (13.5) | 227 (9.2) |  |
| Any prenatal tobacco use |  |  | **<0.001** |
| No | 7,025 (93.6) | 2,066 (90.1) |  |
| Yes | 477 (6.4) | 228 (9.9) |  |
| Clinical Characteristics | | | |
| Early pregnancy BMI |  |  | **0.012** |
| BMI <30 kg/m^2^ | 4,055 (54.1) | 1,193 (51.1) |  |
| BMI ≥30 kg/m^2^ | 3,447 (45.9) | 1,143 (48.9) |  |
| Gestational age at delivery |  |  | **<0.001** |
| Gestational age, weeks | 39.3 (2.1) | 37.6 (6.1) |  |

Data presented as mean (standard deviation) for continuous variables or n (percent) for categorical variables; Bold p-value indicates statistical significance; Missing data for participants excluded from the final analyses ranged from n=17 to n=1,793, with the diet variable having the most missing data; Abbreviations: BMI=body mass index; LTPA=leisure-time physical activity
