# Supplementary material for: Yoga as a form of leisure-time physical activity and pregnancy health outcomes
Source: BMC Pregnancy Childbirth. 2026 Feb 6;26:252. doi: 10.1186/s12884-026-08659-4 (PMC12977424; doi:10.1186/s12884-026-08659-4)
Supplement: Supplementary file 2 — Supplementary Material 2. [file 12884_2026_8659_MOESM2_ESM.docx]

**Supplemental Table 2.** Frequency and Relative Risk of Experiencing an Adverse Pregnancy Outcome by Yoga Group

| **Outcome** | **Yoga Group** | **Total N** | **Percent with**  **Outcomes** | **Model 1**  **RR (95% CI)** | **p-value** | **Model 2**  **RR (95% CI)** | **p-value** | **Model 3**  **RR (95% CI)** | **p-value** |
| --- | --- | --- | --- | --- | --- | --- | --- | --- | --- |
| **Composite APO** | None | 6,237 | 38.3% | 1.0 (Reference) | - | 1.0 (Reference) | - | 1.0 (Reference) | - |
|  | Low | 464 | 34.1% | 0.89 (0.78-1.01) | 0.080 | 0.97 (0.85-1.10) | 0.615 | 0.96 (0.84-1.10) | 0.572 |
|  | Some | 558 | 30.8%% | 0.81 (0.71-0.92) | **0.001** | 0.87 (0.76-0.99) | **0.030^a^** | 0.86 (0.76-0.98) | **0.025^a^** |
|  | High | 243 | 26.8% | 0.70 (0.57-0.86) | **0.001** | 0.78 (0.63-0.97) | **0.024^a^** | 0.78 (0.63-0.96) | **0.020^a^** |
|  |  |  |  | **p-trend** | **<0.001** | **p-trend** | **0.003** | **p-trend** | **0.002** |
| **HDP^b^** | None | 6,029 | 24.1% | 1.0 (Reference) | - | 1.0 (Reference) | - | 1.0 (Reference) | - |
|  | Low | 452 | 22.4% | 0.93 (0.78-1.11) | 0.413 | 0.99 (0.83-1.18) | 0.936 | 0.99 (0.83-1.18) | 0.908 |
|  | Some | 552 | 19.8% | 0.82 (0.69-0.98) | **0.026** | 0.85 (0.72-1.02) | 0.077 | 0.85 (0.72-1.02) | 0.066 |
|  | High | 238 | 18.9% | 0.79 (0.60-1.03) | 0.076 | 0.87 (0.67-1.14) | 0.318 | 0.87 (0.66-1.13) | 0.286 |
|  |  |  |  | **p-trend** | **0.005** | **p-trend** | 0.069 | **p-trend** | 0.056 |
| **PTB** | None | 6,227 | 8.3% | 1.0 (Reference) | - | 1.0 (Reference) | - | 1.0 (Reference) | - |
|  | Low | 464 | 6.5% | 0.78 (0.55-1.11) | 0.169 | 0.91 (0.64-1.31) | 0.621 | 0.92 (0.64-1.32) | 0.641 |
|  | Some | 557 | 6.3% | 0.76 (0.54-1.05) | 0.102 | 0.87 (0.62-1.22) | 0.416 | 0.87 (0.62-1.22) | 0.424 |
|  | High | 242 | 2.9% | 0.35 (0.17-0.73) | **0.005** | 0.42 (0.20-0.87) | **0.020** | 0.42 (0.20-0.87) | **0.020** |
|  |  |  |  | **p-trend** | **0.001** | **p-trend** | **0.033** | **p-trend** | **0.028** |
| **GDM^b^** | None | 6,119 | 4.5% | 1.0 (Reference) | - | 1.0 (Reference) | - | 1.0 (Reference) | - |
|  | Low | 460 | 3.3% | 0.72 (0.43-1.2) | 0.208 | 0.79 (0.47-1.31) | 0.362 | 0.80 (0.48-1.33) | 0.383 |
|  | Some | 556 | 2.9% | 0.64 (0.39-1.05) | 0.074 | 0.66 (0.41-1.09) | 0.107 | 0.68 (0.42-1.13) | 0.137 |
|  | High | 239 | 2.5% | 0.55 (0.25-1.23) | 0.148 | 0.63 (0.28-1.40) | 0.253 | 0.65 (0.29-1.45) | 0.291 |
|  |  |  |  | **p-trend** | **0.014** | **p-trend** | **0.043** | **p-trend** | 0.061 |
| **SGA** | None | 6,182 | 11.0% | 1.0 (Reference) | - | 1.0 (Reference) | - | 1.0 (Reference) | - |
|  | Low | 462 | 8.7% | 0.79 (0.58-1.07) | 0.126 | 0.88 (0.64-1.20) | 0.405 | 0.88 (0.64-1.19) | 0.397 |
|  | Some | 555 | 7.4% | 0.67 (0.50-0.91) | **0.010** | 0.75 (0.55-1.02) | 0.065 | 0.74 (0.55-1.02) | 0.061 |
|  | High | 240 | 5.8% | 0.53 (0.32-0.89) | **0.016** | 0.61 (0.36-1.02) | 0.064 | 0.61 (0.36-1.03) | 0.063 |
|  |  |  |  | **p-trend** | **<0.001** | **p-trend** | **0.005** | **p-trend** | **0.009** |

Model 1 was unadjusted.; Model 2 was adjusted for age, early pregnancy BMI, income, insurance, race, diet, prenatal alcohol use, and prenatal tobacco use; Model 3 was adjusted for Model 2 + LTPA; **^a^** by a p-value indicates age, income, and race were not included as adjustment variables due to convergence issues within the general linear model; ^b^ by an outcome indicates that participants were not included in analyses due to having a preexisting condition prior to pregnancy; Bold p-value indicates statistical significance; Abbreviations: RR=relative risk, 95% CI=95% confidence interval, APO=adverse pregnancy outcome, PTB=preterm birth, SGA=small-for-gestational age infants, HDP=hypertensive disorder of pregnancy, and GDM=gestational diabetes mellitus
