# Supplementary material for: Yoga as a form of leisure-time physical activity and pregnancy health outcomes
Source: BMC Pregnancy Childbirth. 2026 Feb 6;26:252. doi: 10.1186/s12884-026-08659-4 (PMC12977424; doi:10.1186/s12884-026-08659-4)
Supplement: Supplementary file 3 — Supplementary Material 3. [file 12884_2026_8659_MOESM3_ESM.docx]

**Supplemental Table 3.** Fully Adjusted Associations between Yoga and Pregnancy Outcomes Including Participants with Missing Dietary Data

| **Outcome** | **Yoga Group** | **Total N** | **Percent of Events** | **Fully Adjusted Model with Diet Removed**  **RR (95% CI)** | **p-value** |
| --- | --- | --- | --- | --- | --- |
| **Composite APO** | None | 7,441 | 38.6% | 1.0 (Reference) | - |
|  | Low | 519 | 34.3% | 0.95 (0.84-1.08) | 0.436 |
|  | Some | 629 | 29.9% | 0.83 (0.73-0.94) | **0.004^a^** |
|  | High | 281 | 28.1% | 0.80 (0.66-0.97) | **0.021^a^** |
|  |  |  |  | **p-trend** | **<0.001** |
| **HDP^b^** | None | 7,175 | 24.1% | 1.0 (Reference) | - |
|  | Low | 505 | 23.4% | 1.03 (0.88-1.21) | 0.693 |
|  | Some | 621 | 18.5% | 0.81 (0.69-0.96) | **0.014** |
|  | High | 275 | 18.6% | 0.84 (0.65-1.08) | 0.177 |
|  |  |  |  | **p-trend** | **0.017** |
| **PTB** | None | 7,428 | 8.8% | 1.0 (Reference) | - |
|  | Low | 519 | 6.2% | 0.83 (0.59-1.17) | 0.290 |
|  | Some | 628 | 5.9% | 0.79 (0.57-1.10) | 0.570 |
|  | High | 280 | 4.3% | 0.58 (0.33-1.02) | 0.332 |
|  |  |  |  | **p-trend** | **0.016** |
| **GDM^b^** | None | 7,290 | 4.6% | 1.0 (Reference) | - |
|  | Low | 512 | 3.3% | 0.77 (0.48-1.25) | 0.296 |
|  | Some | 627 | 2.9% | 0.65 (0.40-1.03) | 0.068 |
|  | High | 276 | 2.2% | 0.54 (0.24-1.19) | 0.125 |
|  |  |  |  | **p-trend** | **0.014** |
| **SGA** | None | 7,367 | 11.2% | 1.0 (Reference) | - |
|  | Low | 516 | 9.1% | 0.92 (0.69-1.22) | 0.564 |
|  | Some | 625 | 7.5% | 0.76 (0.57-1.09) | 0.066 |
|  | High | 277 | 7.2% | 0.77 (0.50-1.18) | 0.233 |
|  |  |  |  | **p-trend** | **0.035** |
| **Inadequate GWG** | None | 969 | 13.7%% | 1.0 (Reference) | - |
|  | Low | 37 | 7.3% | 0.49 (0.33-0.71) | **<0.001** |
|  | Some | 70 | 11.5% | 0.79 (0.58-1.06) | 0.119 |
|  | High | 31 | 11.8% | 1.00 (0.64-1.57) | 0.996 |
|  |  |  |  | **p-trend** | 0.079 |
| **Adequate GWG** | None | 1,365 | 19.3% | 1.0 (Reference) | - |
|  | Low | 134 | 26.6% | 1.0 (Reference) | - |
|  | Some | 164 | 27.0% | 1.0 (Reference) | - |
|  | High | 60 | 22.9% | 1.0 (Reference) | - |
| **Excessive GWG** | None | 4,742 | 67.0% | 1.0 (Reference) | - |
|  | Low | 333 | 66.1% | 0.72 (0.58-0.89) | **0.003** |
|  | Some | 374 | 61.5% | 0.68 (0.55-0.83) | **0.000** |
|  | High | 171 | 65.2% | 0.85 (0.63-1.15) | 0.299 |
|  |  |  |  | **p-trend** | **<0.001** |

Fully adjusted models were adjusted for age, early pregnancy BMI, income, insurance, race, prenatal alcohol use, and prenatal tobacco use, and LTPA (note: diet was not included as a covariate in these models); **^a^**indicates age, income, and race were not included as adjustment variables due to convergence issues within the general linear model; ^b^indicates that participants were not included in analyses due to having a preexisting condition prior to pregnancy; Bold p-value indicates statistical significance; Abbreviations: RR=relative risk, 95% CI=95% confidence interval, APO=adverse pregnancy outcome, HDP=hypertensive disorder of pregnancy, PTB=preterm birth, GDM=gestational diabetes mellitus, SGA=small-for-gestational age infants, and GWG=gestational weight gain
