# Supplementary material for: Yoga as a form of leisure-time physical activity and pregnancy health outcomes
Source: BMC Pregnancy Childbirth. 2026 Feb 6;26:252. doi: 10.1186/s12884-026-08659-4 (PMC12977424; doi:10.1186/s12884-026-08659-4)
Supplement: Supplementary file 4 — Supplementary Material 4. [file 12884_2026_8659_MOESM4_ESM.docx]

**Supplemental Table 4.** Fully Adjusted Associations of Yoga Consistency with Pregnancy Outcomes

| **Outcome** | **Yoga Exposure Group** | **Total N** | | **Percent of Events** | **Fully Adjusted Model with 3 Yoga Groups**  **RR (95% CI)** | **p-value** |
| --- | --- | --- | --- | --- | --- | --- |
| **Composite APO** | None | 6,237 | | 38.3% | 1.0 (Reference) | - |
|  | Inconsistent | 1,068 | | 31.7% | 0.89 (0.81-0.98) | **0.018^a^** |
|  | Consistent | 197 | | 28.9% | 0.85 (0.68-1.06) | 0.143**^a^** |
|  |  |  | |  | **p-trend** | **0.008** |
| **HDP^b^** | None | | 6,029 | 24.1% | 1.0 (Reference) | - |
|  | Inconsistent | | 1,049 | 20.4% | 0.89 (0.78-1.02) | 0.089 |
|  | Consistent | | 193 | 21.2% | 0.96 (0.73-1.27) | 0.783 |
|  |  | |  |  | **p-trend** | 0.164 |
| **PTB** | None | | 6,227 | 8.3% | 1.0 (Reference) | - |
|  | Inconsistent | | 1,066 | 6.4% | 0.89 (0.69-1.15) | 0.381 |
|  | Consistent | | 197 | 2.0% | 0.29 (0.11-0.77) | **0.013** |
|  |  | |  |  | **p-trend** | **0.020** |
| **GDM^b^** | None | | 6,119 | 4.5% | 1.0 (Reference) | - |
|  | Inconsistent | | 1,061 | 2.9% | 0.70 (0.49-1.02) | 0.064 |
|  | Consistent | | 194 | 3.1% | 0.82 (0.37-1.82) | 0.625 |
|  |  | |  |  | **p-trend** | 0.096 |
| **SGA** | None | | 5,182 | 11.0% | 1.0 (Reference) | - |
|  | Inconsistent | | 1,063 | 7.5% | 0.76 (0.60-0.96) | **0.020** |
|  | Consistent | | 194 | 7.7% | 0.82 (0.50-1.35) | 0.430 |
|  |  | |  |  | **p-trend** | **0.030** |
| **Inadequate GWG** | None | | 778 | 13.1% | 1.0 (Reference) | - |
|  | Inconsistent | | 103 | 9.9% | 0.71 (0.55-0.92) | **0.010** |
|  | Consistent | | 29 | 10.2% | 0.73 (0.43-1.26) | 0.262 |
|  |  | |  |  | **p-trend** | **0.012** |
| **Adequate GWG** | None | | 1,164 | 19.5% | 1.0 (Reference) | - |
|  | Inconsistent | | 271 | 26.2% | 1.0 (Reference) | - |
|  | Consistent | | 53 | 28.5% | 1.0 (Reference) | - |
| **Excessive GWG** | None | | 4,016 | 67.4% | 1.0 (Reference) | - |
|  | Inconsistent | | 662 | 63.9% | 0.74 (0.63-0.88) | **<0.001** |
|  | Consistent | | 114 | 61.3% | 0.66 (0.47-0.93) | **0.017** |
|  |  | |  |  | **p-trend** | **<0.001** |

Yoga groups were defined as: None (no yoga in any trimester), Inconsistent (yoga is reported in some but not all available trimesters), and Consistent Exposure (yoga is reported in all available trimesters); Fully adjusted models were adjusted for age, early pregnancy BMI, income, insurance, race, diet, prenatal alcohol use, and prenatal tobacco use, and LTPA; **^a^**indicates age, income, and race were not included as adjustment variables due to convergence issues within the general linear model; ^b^indicates that participants were not included in analyses due to having a preexisting condition prior to pregnancy; Bold p-value indicates statistical significance; Abbreviations: RR=relative risk, 95% CI=95% confidence interval, APO=adverse pregnancy outcome, HDP=hypertensive disorder of pregnancy, PTB=preterm birth, GDM=gestational diabetes mellitus, SGA=small-for-gestational age infants, and GWG=gestational weight gain
